# Assessing Effectiveness and Cost-Benefit of the Trinity Hospital Twin City Fit For Life Program for Weight Loss and Diabetes Prevention in a Rural Midwestern Town

**DOI:** 10.5888/pcd15.170479

**Published:** 2018-08-02

**Authors:** Timothy McKnight, Jennifer R. Demuth, Natalie Wilson, Jonathon P. Leider, Alana Knudson

**Affiliations:** 1 Trinity Hospital Twin City, Dennison, Ohio; 2 Ohio University, Voinovich School of Leadership and Public Affairs, Athens, Ohio; 3 Johns Hopkins University Bloomberg School of Public Health, Health Policy and Management, Baltimore, Maryland; 4 National Opinion Research Center, Public Health, Chicago, Illinois

## Abstract

**Introduction:**

Obesity is a top public health priority in the United States. This article reports on the Fit For Life (FFL) health education program designed to address the determinants of obesity in rural settings and help participants lose weight.

**Purpose and Objectives:**

We evaluated the implementation of the original FFL program, a replication program, and a diabetes-focused program.

**Intervention Approach:**

The original FFL program (2006 to 2012) was a 12-week session of classes meeting once weekly. Lecture topics included stress management, nutrition, healthy eating, reading food labels, fitness, disease prevention, and healthy aging. The replication program, conducted in 4 locations from 2012 to 2015, helped determine if the FFL program could be implemented on a larger scale, with outcomes similar to the original program. The longer, more-intensive FFL diabetes prevention program, conducted in 2016 and 2017, sought to reduce the number of rural adults at risk for diabetes.

**Evaluation Methods:**

We evaluated FFL participation and outcome data from 2009 through first quarter 2017. We calculated rates of course participation and completion and measured changes in several health indicators. We constructed a linear regression model to examine the impact of health behaviors on weight loss and calculated program cost-effectiveness.

**Results:**

From 2009 to 2017, FFL was delivered to over 1,200 people; 82% of participants completed the program. Completing participants lost an average of 2.7 kg or 3% of their total weight. Overall, 68% of participants said they exercised more per week at the end of the program than at the beginning. Estimated cost per kilogram lost for replication sites was between $73 and $101 for original FFL, in line with other programs. The more resource-intensive diabetes prevention program cost per kilogram lost was $151 to $171.

**Implications for Public Health Practice:**

Weight loss and lifestyle management are major ways to counteract obesity. Improving program options, especially in rural locales, should be a key policy priority. These programs should be considered for population-based expansion, perhaps by health departments or public–private health care consortiums.

## Introduction

Obesity and other lifestyle-related chronic diseases are a top US health policy priority (1–3). Obesity costs the United States at least $147 billion annually in direct healthcare costs and $4 billion in other costs, including lost productivity (4–6). Obesity dramatically affects the largest areas of spending — on diabetes ($237 billion) and ischemic heart disease ($88 billion) (7). Obesity and overweight are drivers of heart disease–related and other circulatory-related deaths, which are now the leading cause of death in the United States (8).

The policy priority for reducing obesity and improving heart health has driven the creation of initiatives, both large and small (9–12). From the Million Hearts Initiative to proprietary health improvement programs, the United States is now awash in potential interventions to fight obesity and improve heart health. A major class of programs includes lifestyle interventions, also known as behavioral weight loss interventions or weight management programs, whereby individuals are educated and motivated to improve their own health through behavior change (13). These programs usually include modifications to diet and exercise regimens. A 2017 systematic review from Sun et al showed that 64 studies of dietary interventions all demonstrated statistically significant weight loss, ranging from −1.17 kg to −3.15 kg (13). Internet- or computer-based interventions also showed some promise as an effective modality (14). That review included few robust findings in rural settings. A recent study by Radcliff et al found better cost-effectiveness for telephone-based programs ($33/kg) versus face-to-face programs ($47/kg) in a rural setting, although face-to-face participants lost more weight, on average (15).

This article aims to contribute to the gap in the literature on the adoption of rural weight loss programs and lifestyle interventions. It relays the experience of a lifestyle intervention delivered in person, specifically focused on assessing the effectiveness, replicability, and cost-effectiveness of lifestyle changes for weight loss, as implemented in a rural Appalachian county.

## Purpose and Objectives

Tuscarawas County is an Appalachian county located in east central Ohio. The county is home to about 92,000 people, of whom 97% are non-Hispanic white. About 86% of the population have graduated from high school, and 14% have a bachelor's degree or higher (compared with 26% in Ohio and 30% nationwide). About 9% of Tuscarawas County residents do not have health insurance. The median household income in the county is just over $45,000 (compared with $49,000 statewide and $54,000 nationwide). Thirteen percent of the population lives in poverty (16). Tuscarawas County ranks in the middle of the pack for health outcomes and behaviors — about 35% of residents have obesity, compared with 30% statewide (17).

In 2006, Trinity Hospital Twin City received funding from the US Department of Health and Human Services (HHS), Health Resources and Services Administration (HRSA), Office of Rural Health Policy (ORHP) Rural Health Outreach Grant Program to offer the Fit For Life (FFL) program in Tuscarawas County, Ohio. IRB approval was not sought because analyses were performed on de-identified secondary data.

After the pilot program (2006–2009), HRSA supported growth of the FFL program and systematic data collection on health outcomes associated with the program. Health outcomes were pre and post measures of weight, total cholesterol, high-density lipoprotein (HDL), low-density lipoprotein (LDL), triglycerides, and blood pressure (systolic/diastolic). In 2012, for the replication study, pre- and post-program surveys were administered to participants, focusing largely on individual health behaviors. In 2015, with the diabetes prevention (DP) program, HbA_1c_ was added as a pre/post measurement and a repeat measurement was added 3 months after conclusion of the 12-week program.

## Intervention Approach

### Original FFL program

Trinity Hospital Twin City’s original FFL Program (from 2006 to 2012) provided health education and promotion through FFL curricula on wellness and disease prevention in classes designed specifically for adult men and women. In a 12-week session of classes that met once weekly for 90 minutes, students heard lectures on stress management, nutrition, healthy eating, reading food labels, fitness, disease prevention, healthy aging, and more (18). In the program, lectures and health education were provided by the Program Director (a medical doctor and Board Certified Family Practitioner). Additional FFL professional presenters often included chiropractors, health coaches, fitness instructors, and wellness educators. In 2012, HRSA provided funding to begin FFL replication studies; in 2015, HRSA funded a diabetes-specific prevention program.

### Replication program

The primary aim of the Fit For Life Replication Project was to assess whether the original program could be implemented on a larger scale with similar clinical outcomes. The replication project sought to provide a multi-agency approach to reduce the number of adults with overweight or obesity in rural Tuscarawas County, Ohio and replication sites in the bordering Appalachian counties of Carroll, Harrison, Holmes, and Jefferson. Effectiveness and sustainability were primary outcomes of interest, leading to an increased number of sites and, ultimately, individuals served by FFL. The curriculum taught at the replication sites was the same as taught in the original FFL project, though different trainers were used. The replication project used a train-the-trainer approach, with 4 replication sites offering services in their respective counties after being trained by staff from the original site. In the first year, 2 half-day trainings were held, and for the last 2 years, 1 half-day training was held each year. A standardized trainer’s manual with a step-by-step guide about how to set up an FFL program was developed. Evaluators participated in the replication site courses to ensure fidelity to the curriculum. Two replication sites left after year 1 of the 3-year project and were replaced in year 2 by other partners. The sites that ceased operation of FFL did so because of staffing constraints and resourcing issues. Of the 4 replication sites, 2 were physician-led, 1 was led by a master’s level health educator, and 1 was co-led by a chiropractor and a master’s level health educator. All other aspects of the program remained the same.

### Diabetes prevention program

The most recent evolution of FFL is in prediabetes management. Interventions for prediabetes are critical, but consistent evidence is lacking about the effectiveness of such programs (19,20). The FFL Diabetes Prevention (DP) program was designed as a time- and resource-intensive intervention to help those at risk for type 2 diabetes, as defined by hemoglobin A_1c_ (HbA_1c_) ≥5.7% or a BMI ≥25). The aim of the DP program was to reduce the number of adults at risk for diabetes – measured through weight loss or BMI change and changes to HbA_1c_. Like the original FFL program, this curriculum also involved the foundational 12-week session that met once a week for about 90 minutes, and it added 3 classes that met once a month. FFL DP students also had access to 3 individual counseling sessions and 3 personal training sessions at no additional cost. This program was conducted at the original FFL site in Tuscarawas County, beginning in late 2015.

## Evaluation Methods

### Data management

General health outcomes data were aggregated, by year, for FFL participants. The health outcomes data set and the health behavior survey data set were linked through a participant ID number. No names or contact information were included in analytic data sets. Outcomes were organized by FFL project type: original FFL (classes ending in 2010–2012), FFL replication (2013–2015), and FFL DP (2016 and 2017).

### Measures 

The primary outcome of interest was change in weight. Other outcomes were changes in BMI (measured as weight in kg divided by height in m^2^), cholesterol, triglycerides, blood pressure, and, for the DP program, HbA_1c_. Individual health behaviors were assessed, including intake of sugar-sweetened beverages, use of Nutrition Facts labels, and exercise (days per week), all of primary interest in this analysis. Additional variables examined were daily servings of fruit and vegetables, daily servings of whole grains, avoidance of *trans* fats, and how often the individual ate outside the home. Age and gender were also accounted for. Anyone who signed up for the program became a participant; no exclusion criteria were used. Completion of the program was defined as a participant attending 7 or more classes (minimum dosing requirement) and not missing more than 3 classes in a row (sequential requirement). Completions were only counted once per person for the years analyzed.

### Analytic / Evaluation approach

Measures of change were of primary interest in the FFL program, the replication project, and the DP project. Data from the first years of the post-pilot project (2009 through 2012) are presented as summary measures only, because the program was still in development and only historical summary measures existed (ie, aggregate information only, no individual record data sets remain). For 2013–2017, individual-level pre/post records are available. We describe changes to health outcomes, focusing especially on weight loss and, for the DP program, changes to HbA_1c_ measures pre and post FFL. Outcomes by replication site were examined and pairwise mean comparisons were conducted to examine site outcome variations, by using the Tukey test for multiple comparisons. Similar comparisons were conducted for years 1 and 2 of the DP program.

In addition to descriptive analyses, we constructed a linear regression model by using robust variance estimators. Weight change in kilograms was the primary dependent variable. Independent variables included baseline information: weight, age, gender, days of exercise per week, consumption of any sugar-sweetened beverages in a week (yes/no), and whether the individual used Nutrition Facts labels “most of the time” or “always.” The model also controlled for program iteration and FFL site. Other variables were examined for inclusion, including baseline daily servings of fruit and vegetables, daily servings of whole grains, avoidance of *trans* fats, and how often the individual ate outside the home. These variables were excluded from the final model because of high correlation with other covariates (as measured through variance inflation factor analysis). 

The analytic data set used in the regression consisted of pre/post measurements from participants who completed FFL, the replication project, or the DP project. Respondents were excluded from overall analyses if they did not complete the program. We also conducted a cost-effectiveness analysis in an effort to quantify potential utility of FFL if replicated more broadly. HRSA and Trinity Hospital Twin City have made substantial investments in the program’s creation since 2006. However, replication program costs (ie, educational materials, site, staffing, and incentives) are relatively modest. We conducted cost-effectiveness analyses for potential replication sites for both original FFL and FFL DP, using cost per kilogram (kg) lost as the primary outcome of interest. This is in line with previous examinations of the cost-effectiveness of weight management interventions (13). We estimated cost-effectiveness by number of students graduated, assuming approximately 20% of FFL participants drop out. This is based on programmatic data for FFL in 2013–2017. Costs were based on actual program costs from 2016–2017. Using results from the original FFL and FFL DP alongside estimated cost for delivering the service, we estimated cost per kg lost among completing participants for class sizes of 30 and 50. Size estimates are based on average class size (ie, 30) and the largest size we believe to be reasonable (ie, 50). We did not include non-service delivery costs (eg, travel, conferences, program administrator) as these costs are not necessary to deliver the FFL program in replication sites. Additionally, we did not include the cost of the curriculum, as it is a one-time purchase. For the original FFL session, we used site data from the replication study in 2012–2015. Data were managed and analyzed in Stata 13.1 (StataCorp LP, College Station, Texas).

## Results

### Demographics and program characteristics

Between 2009 and 2017, FFL was delivered to over 1,200 people in 3 distinct iterations. First, between 2009 and 2012, FFL was delivered at its primary site. Second, between 2012 and 2015, FFL was delivered at 4 replication sites. Third, in 2016 and 2017, FFL was delivered at its primary site in an expanded format to individuals at risk for diabetes (the DP program). Overall, more women than men participated – 74% versus 26% (Table 1). Most participants were aged between 45 and 64 years. Overall, about 43% of respondents said their household income was below $51,000. Among the FFL DP participants who completed bloodwork (n = 108 in 2016 and 2017), the mean and median HbA_1C_ value was 5.7. The 25^th^ and 75^th^ percentiles were 5.4 and 6.0, respectively. The mean and median BMI were 36 and 34, respectively.

**Table 1:** Demographics of Participants in the Trinity Hospital Twin City Fit For Life Weight Loss and Diabetes Prevention Programs, Rural Ohio, 2009–2017

Demographic	Original FFL				Replication Study			Diabetes Prevention Program		Total
Year	2009	2010	2011	2012	2013	2014	2015	2016^a^	2017^a^	–
Participants (n)	134	335	116	71	86	147	195	55	92	1231
Returned Surveys (n)	127	305	107	64	68	97	85	42	70	965
**Gender (%)**										
Female	71	76	63	73	74	82	69	70	83	74
Male	28	23	36	27	26	18	31	30	17	26
Missing	1	1	1	0	0	0	0	0	0	0
**Age Group, y (%)**										
18–44	26	25	35	17	10	19	13	15	18	22
45–64	58	67	51	67	60	60	68	54	66	62
≥65	16	8	14	16	30	21	19	30	16	16
Missing	0	0	0	0	0	0	0	0	0	0
**Household Income, $ (%)**										
0–30,999	25	12	17	14	11	11	14	27	12	15
31,000–50,999	24	24	27	27	31	38	26	44	37	28
≥51,000	36	56	48	48	53	41	51	26	46	48
Declined to answer	14	9	8	11	4	9	9	3	4	9

Abbreviation: FFL, Fit For Life.

a Fit For Life Diabetes Prevention Program, full course.

Since 2009, FFL completion rates have been tracked by session and delivery site. Table 2 shows overall completion rates and program outcomes for 2009, 2013, and 2017. In total, 82% of participants completed FFL in 2009–2017, almost 1,100 in total. On average, participants lost 2.7 kg, or 3% of their total weight, from the beginning of the 12-week program to the end, and 1.25 points of BMI. This amount increased during the course of the program, with average weight loss increasing from 1.4 kg in 2009–2012 to 2.7 kg in 2013–2015 to 6.1 and 5.0 kg in 2016 and 2017, respectively. 

**Table 2:** Health Outcomes for Select Years, Trinity Hospital Twin City Fit For Life Weight Loss and Diabetes Prevention Programs, Rural Ohio, 2009–2017

Health Measure	2009	2013	2017
No. of Participants	134	86	92
% completing	91	79	78
% of completions participating in final measurements	80	100	99
% of class participating in final laboratory measurements	70	96	97
**Weight loss, kg**			
Total weight lost	173	191	302
Avg. loss	1.2	2.8	4.3
Avg. % of weight loss	3	3	4
**BMI averages**			
BMI lost	1.1	1.0	1.6
% of BMI	2	3	4
**Blood pressure, average change**			
Systolic	3.5	6.0	2.2
Diastolic	−2.7^a^	0.2	3.3
**Lipids, average change**			
Cholesterol	10.3	11.0	7
HDL	0.0	−0.6^ a^	−0.7^ a^
LDL	8.1	8.7	4.8
Triglycerides	5.6	17.3	14

Abbreviations: BMI, body mass index; HDL, high-density lipoprotein; LDL, low-density lipoprotein.

a Negative numbers reflect gains.

During 2013–2015, participants’ weight changed:
20% lost more than 5 kg
29% lost between 2.5 and 5 kg
34% lost between 0 kg and 2.5 kg
15% gained between 0 and 2.5 kg, and
2% gained more than 2.5 kg.

Overall, 52% of individuals in 2013–2015 lost 3% or more of their bodyweight, and 27% lost 5% or more. Comparatively, for the 2016 and 2017 FFL DP, 74% of individuals lost 3% or more of their total weight, and 48% lost 5% or more.

In FFL DP during 2016 and 2017, participants’ weight changed:
41% lost more than 5.0 kg
28% lost between 2.5 kg and 5.0 kg
20% lost between 0 kg and 2.5 kg
9% gained between 0 and 2.5 kg, and
2% gained more than 2.5 kg.

At the 3-month post-intervention follow-up, FFL DP participants (n = 62) had lost 6.1 kg on average, representing further weight loss after the conclusion of the 12-week program (median = 5.5 kg). The median weight regain from the end of the 12-month program to 3-month follow-up was 0 kg. Among those further losing weight (n = 31), the average was an additional 3.3 kg. Among those regaining weight (n = 31), the gain was 2.0 kg, representing a net decrease of 1.3 kg from the beginning of the program for these participants.

On average, not only weight, but also blood pressure, total cholesterol, and triglycerides were reduced. Between original FFL and FFL DP, the change in systolic blood pressure was not substantial or statistically different (−4.4 vs −3.4 points, *P* = .6), although diastolic was (−0.94 vs −3.5 points, *P* = .02). Cholesterol (total, HDL, and LDL) and triglycerides were not statistically different between the 2 programs.

The replication study included the primary site and 4 additional sites. After the first year of the replication study, 2 sites discontinued the program and 1 new site began offering the program. The primary site had 253 total participants during the replication project period and Sites 1, 2, 3, and 4 had 13, 12, 84, and 61 participants, respectively. Course completion for the primary site was 83% and for Sites 1, 2, 3, and 4 completion rates were 85%, 75%, 75%, and 62%, respectively.

**Figure 1:**
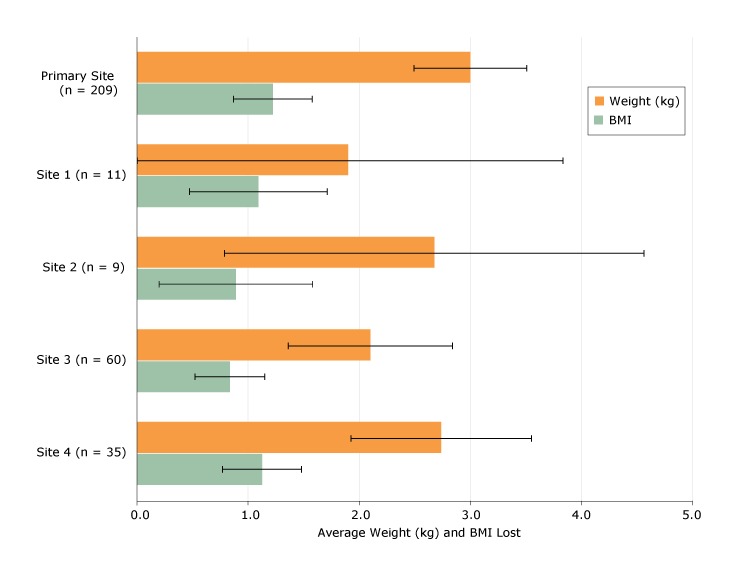
Weight and BMI changes by primary and ancillary replication sites, Trinity Hospital Twin City FFL Program, 2013–2015. Outcomes are shown as average weight (kg) and BMI lost, with 95% confidence intervals. Abbreviations: BMI, body mass index; FFL, Fit For Life Program. **Table:** 

Weight change was comparable across sites (Figure 1). Although the primary site showed a larger average change in weight during the replication study period (mean, 3.0 kg, standard error [SE], 0.25 kg), these differences were not statistically significant compared with all replication sites (mean = 2.3, SE = 0.26, *P* = .08). All sites had participants whose weight change was negative. The null hypothesis was that the program would have no effect on weight change. 

Setting	No.	Average Weight Lost (95% CI), kg	Average BMI Lost (95% CI)
Primary site	209	3.0 (2.5–3.5)	1.2 (0.8–1.6)
Site 1	11	1.9 (0–3.8)	1.1 (0.5–1.7)
Site 2	9	2.7 (0.8–4.6)	0.9 (0.2–1.6)
Site 3	30	2.1 (1.4–2.8)	0.8 (0.5–1.1)
Site 4	65	2.7 (1.9–3.5)	1.1 (0.7–1.5)

Participants at the primary site lost 3 kg on average (n = 187 completing measurements, standard deviation [SD] = 3.47, *P* < .001).
At Site 1, participants lost 1.9 kg on average (n = 11, SD = 3.2, *P* = .08)
At Site 2, participants lost 2.7 kg on average (n = 9, SD = 2.9, *P* = .023)
At Site 3, participants lost 2.1 kg on average (n = 52, SD = 2.7, *P* < .001)
At Site 4, participants lost 2.7 kg on average (n = 35, SD = 0.4, *P* < .001).

**Figure 2:**
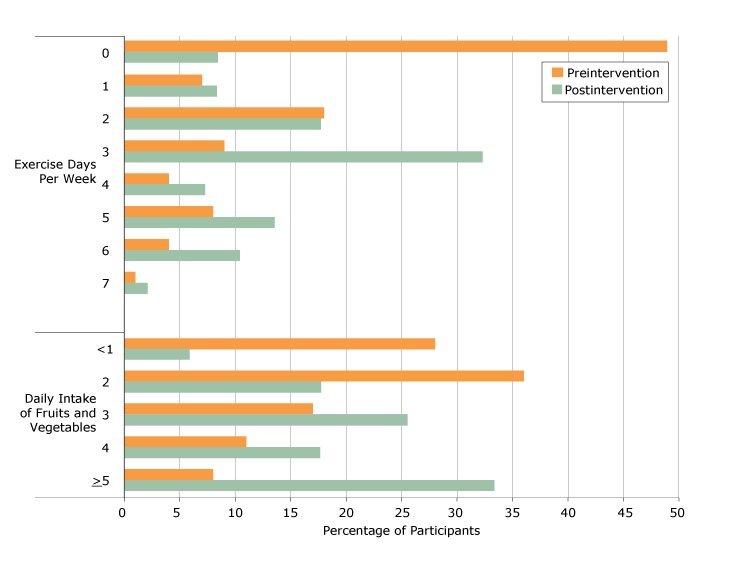
Health behavior outcomes among replication study sites and diabetes prevention site, Trinity Hospital Twin City FFL Program, 2013–2015.

Improvements in health behaviors were noted at the replication sites and in the FFL DP. At the beginning of the 12-week program, 46% of participants who completed the survey (n = 347) said they exercised 0 times per week, 37% said 1–3 times, and 17% said 4–7 times. At the end of the program, 11% said they exercised 0 times per week, 49% said they exercised 1–3 times, and 39% said they exercised more than 4 times. Overall, 68% of participants said they exercised more per week at the end of the program than at the beginning. Among these individuals, average increase in weekly exercise was 2.6 times per week (median 2, SD 1.4). Similar positive changes in health behaviors were observed with eating fruit and vegetables (67% improved habit), eating whole grains (54%), avoiding *trans* fats (54%), avoiding high fructose corn syrup (59%), and using Nutrition Facts labels (56%, Figure 2). Among DP program participants, 54% saw HbA_1c_ values improve about 1.5% on average. Overall, about 34% of participants whose HbA_1c_ was 5.7% or higher at the beginning of the FFL diabetes prevention program decreased it to normal levels by the end of the program.

We conducted a linear regression, modeling weight loss in kgs for participants in the replication study and the DP group (Table 3). After controlling for weight and age, gender, days of exercise by the end of the program period, consumption of sugar-sweetened beverages by the end of the program period, using nutrition labels by the end of the program period, and the program type (original FFL versus FFL DP) all had statistically significant effects on weight loss. Each additional day of exercise per week was associated with a 0.4 kg decrease in weight over the 12-week period, all else equal (*P* = .001). Being in the FFL DP program was associated with greater weight loss compared with the original FFL program (2.4 kg, *P* < .001).

**Table 3:** Regression Results, Trinity Hospital Twin City Fit For Life Weight Loss and Diabetes Prevention Programs, Rural Ohio, 2009–2017

Variable	Coefficient (weight loss, kg)	95% CI	*P* value
Exercise (days per week)	−0.39	(−0.61 to −0.17)	.001
≥1 sugar-sweetened beverage per week	1.05	(0.21–1.9)	.014
**Use Nutrition Facts panel/label**			
Never/Rarely/Sometimes	[Reference]		
Most of the time/always	−1.23	(−2.18 to −0.28)	.011
**FFL program type**			
Original/replication study	[Reference]		
Diabetes prevention	−2.44	(−3.51 to −1.37)	<.001
**Controls**			
Weight (kg)	−0.05	(−0.06 to −0.03)	<.001
Age (years)	0.05	(0.02–0.08)	.003
Female	1.06	(0.15–1.97)	.022

### Estimating cost-benefit 

We estimate program costs at $5,750 for original FFL and $7,188 for the longer FFL DP (Table 4). This is equivalent to $192 per participant in a original FFL program with a class size of 30 participants completing the program (assuming 80% of the original class completes the program). Add to this the variable cost per person (including incentives, workbooks, and other supplies) for the original FFL program, and a class of 30 completing students costs about $261 per person, or $279 per completing student assuming an 80% completion rate. The DP program has somewhat higher fixed costs (for 3 extra sessions), but substantially higher variable costs — as participants had access to personal training sessions and counseling. As such, cost per completed student is significantly higher in the DP program. However, because participants tended to lose substantially more weight on that program, per kg costs were more modest. From a purely cost-effectiveness perspective, original FFL performs better (incremental cost-effectiveness ratio, $178–$184, depending on class size, comparing original FFL with FFL DP). Cost based on the number of completions with a 3%+ and 5%+ weight loss are even higher: $544 per student with weight loss of 3% or more (FFL) and $1,029 (DP), and $1,056 and $1,587 per student with weight loss of 5% or more (FFL and FFL DP, respectively).

**Table 4:** Estimating Cost-Benefit for a Replication Site, Trinity Hospital Twin City Fit For Life Weight Loss and Diabetes Prevention Programs, Rural Ohio, 2009–2017

Cost Factor	Original FFL 3 months (12 sessions)^a^	Diabetes Prevention 6 months (15 sessions)^b^
**Fixed cost ($)**		
Facilitator^c^ ($313 per session)	3,750	4,688
Staff coordinator^c^ ($75 per session)	900	1,125
Space rental ($50 per session)	600	750
Contracted speaker (2 at $250 per class)	500	625
Subtotal (fixed costs)^d^	5,750	7,188
**Variable cost per person ($)^e^**		
Supplies/Workbooks	20	20
Incentives	20	20
Blood draws^f^	30	30
Counseling	–	300
Fitness training	–	100
Subtotal (variable costs)	70	470
**Cost per completed students ($)^g^**		
30 per class	279	827
50 per class	203	731
**Cost per kilogram lost among completed students ($)**		
30 per class	101 (95% CI, 89–117)	170 (95% CI, 146–205)
50 per class	73 (95% CI, 65–85)	151 (95% CI, 129–181)
Average weight loss post intervention (kg)	2.75 (95% CI, 2.4–3.1)	4.8 (95% CI, 4.0–5.7)

Abbreviations: CI, confidence interval; FFL, Fit For Life.

a Assumes 80% completion.

b Provided at a substantial discount to FFL participants.

c Includes 25% fringe benefits.

d Estimates do not include one-time purchase cost of curriculum (approximately $2,000).

e Cost per person assumes students who do not complete course still consume all variable goods/services.

f Provided at discount from the hospital.

g Cost for *c* completed students for *n* total students calculated as (Fixed cost + (variable costs×*n*))÷*c*.

## Implications for Public Health Practice

The FFL programs showed consistent, statistically, and clinically significant weight loss results between 2009 and 2017. Participants in the original FFL program lost 2.7 kg and 3.2% of their BMI on average, and participants in the FFL DP program lost 4.8 kg and 4.8% of BMI. Cholesterol improved (ie, was lower) by 3.6%, blood pressure by 2.3% (systolic) and 1.4% (diastolic), and triglycerides by 4.8% on average. Moreover, participants, on average, said they exercised more, ate more fruits and vegetables, and employed more healthy behaviors after completion of FFL compared with before initiating the program.

Estimates for cost-effectiveness for original FFL ranged between $73 and $101 per kg lost among completing students, contingent on class size. This is in line with findings from a recent meta-analysis on the cost and effectiveness of similar lifestyle interventions (13,15,21–26). The program was lower cost than commercial weight loss interventions, the least expensive of which is around $155 per kg lost (95% CI, $110–$218) and pharmaceutical interventions, the least expensive of which is $204 per kg (21). The FFL program is considerably less expensive and more effective than more expensive interventions (eg, $2,204 per kg lost in the Be Fit Be Well program) (27). However, several interventions were more effective (13) and cost-effective (lowest cost was $34 per kg lost). Additionally, a small number of interventions including technology, like multi-sensor armbands, were more cost-effective ($51/kg) (23).

In the higher-risk and higher-cost population — those in the DP program — estimates of cost effectiveness ranged from $150 to $170 per kg lost, based on class size. As the United States spends $237 billion a year in direct medical care for diabetes (28), FFL DP may be a cost-effective alternative. We posit this may be especially true in rural areas (16). Beyond weight loss or improved heart health, FFL and similar lifestyle interventions have other potential benefits that are more difficult to quantify (22), such as reduced personal healthcare costs from improved health. In the experience of FFL staff, participants often dramatically reduce the number of medications they take after experiencing weight loss and improved heart health. Moreover, healthy eating and lifestyle choices may spill over to a participant’s family and friends.

In our view, cost-benefit and cost-utility analyses of lifestyle interventions and other weight-loss interventions must be expanded to include the broader set of benefits that participants realize (29,30). Although it is beyond the scope of this article to propose alternative measures of cost-benefit analyses, we do support moving beyond facile cost-per-kilogram approaches to evaluation. We recognize the importance of the use of a simple, universal comparator, but, from a policy perspective, do not think that cost-per-kilogram-lost is the best choice.

### Implementing a replication program and a diabetes prevention intervention: lessons learned

The replication of FFL into other jurisdictions was successful; results were relatively consistent across sites, and all experienced reasonable participation rates. However, 2 sites did discontinue participation early on in the process. Circumstances at the sites themselves caused these changes, not the FFL program. In the withdrawal letter from 1 organization, a newly appointed leader cited the need to discontinue participation because of the organization’s small staff and the need to shift priorities to other projects within their jurisdiction; the project was something he “inherited” with the retirement of the prior leader, with whom Trinity Hospital Twin City had an excellent working relationship. The second site that discontinued participation was a critical access hospital. In their withdrawal letter, the CEO indicated a lack of staffing and resources for the program because the doctor who had been trained by Trinity Hospital Twin City was about to take maternity leave. About a year after the critical access hospital discontinued participation in FFL, the hospital experienced significant financial trouble which led to its sale. It is our belief that the hospital’s broader staff shortage and financial situation also played a role in the decision to discontinue. Successful partnerships in the replication phase required good institutional support from partners and ongoing technical assistance and collaboration from the primary site.

We view the FFL DP program as a modest success. Because of the high number of Tuscarawas County adults at risk for diabetes, Trinity Hospital Twin City created FFL DP to target more specifically those at highest risk for diabetes (ie, only adults with prediabetes and high diabetes risk factors such as obesity, family history, and elevated HbA_1c_ levels were accepted into the DP program, as opposed to original FFL, which was open to anyone in the community). HRSA’s funding created the opportunity for the hospital to make the DP program affordable; however, for cost-effectiveness reasons, the program will be sustained beyond grant funding by returning to an adapted original FFL curriculum. The following adaptations will be made to the original FFL as a result of the success of the DP program: there will be continued involvement of the counseling and fitness professionals on a smaller scale by providing some services in-kind during class time and the 3 monthly classes will likely continue after the initial 12-week course is completed. We believe this maximizes sustainability while still using successful components of the DP program.

### Limitations

Analyses presented in this article are subject to numerous questions related to both external and internal validity. Most important is the potential critique of generalizability. Fundamentally, individuals participate in FFL because of some desire to effect behavior change. Although over half of FFL participants said they had “recently” or “this month” resolved to have a healthier lifestyle, FFL participants are inherently a motivated group of individuals or are brought to the class by a friend or family member who is. It seems, then, that FFL participants ought not be generalized to the general public – merely those considering a lifestyle intervention or weight-management program. Measurement error is always a threat to internal validity. To address this, equipment was calibrated, the same equipment was used for all repeated measures, and all clinical diagnostic tests were conducted by certified laboratory staff. A final consideration is post-intervention measurement. Three-month follow-ups were conducted starting in 2016, with about 60% of those who completed the program participating in the follow-up. Long-term weight regain remains a valid concern. Our experience with weight regain is limited by those participating in the study — about half continue to lose weight, and half regained some (although net weight loss remained).

## Conclusion

Trinity Hospital Twin City FFL is one among numerous lifestyle intervention and weight loss programs available in the United States (13). It seeks to harness the motivation of its participants, offering access to health education and nutrition information. It was designed to serve a rural population, and its focus goes beyond diet and exercise, to include health improvement holistically. From a policy perspective, funding these types of weight management and lifestyle interventions, especially in rural jurisdictions, could prove extremely productive and cost-effective. Rather than Medicaid or other publicly subsidized insurance paying to manage multiple chronic conditions or an individual dealing with the health and economic ramifications of diabetes, primary prevention is both the prudential and cost-effective alternative. As such, jurisdictions, especially rural ones, should consider offering weight management and other lifestyle interventions for interested parties. Our work with governmental public health agencies in the replication studies suggests public health departments may be a natural ally of local hospitals and clinics on this particular topic.

Regardless of location or particular curriculum, lifestyle intervention programs and other behavioral interventions must come to form a foundation of universal access to health education and nutrition assistance. The programs are relatively inexpensive and relatively effective (31). Individual behaviors account for 30% of an individual’s health outcomes (32), so cost-effectively motivating individual behavior change must remain a top research and policy priority.
